# CORRIGENDUM

**DOI:** 10.1002/mpr.1800

**Published:** 2019-12-29

1

A correction to

Barriers of mental health treatment utilization among first‐year college students: First cross‐national results from the WHO World Mental Health International College Student Initiative

Volume 28, Issue 2, e1782.

In the June, 2019, issue of this Journal, we published the article above referenced, authored by “David Daniel Ebert, Philippe Mortier, Fanny Kaehlke, Ronny Bruffaerts, Harald Baumeister, Randy P. Auerbach, Jordi Alonso, Gemma Vilagut, Kalina U. Martínez, Christine Lochner, Pim Cuijpers, Ann‐Marie Kuechler, Jennifer Green, Penelope Hasking, Coral Lapsley, Nancy A. Sampson, Ronald C. Kessler”

The author regrets an error regarding her name. “Kalina U. Martínez” should be corrected to “Kalina I. Martínez.”

